# Designing a Useful Lipid Raft Model Membrane for Electrochemical and Surface Analytical Studies

**DOI:** 10.3390/molecules26185483

**Published:** 2021-09-09

**Authors:** Michalina Zaborowska, Damian Dziubak, Dorota Matyszewska, Slawomir Sek, Renata Bilewicz

**Affiliations:** 1 Faculty of Chemistry, University of Warsaw, Pasteura 1, 02093 Warsaw, Poland; mzaborowska@chem.uw.edu.pl; 2 Faculty of Chemistry, Biological and Chemical Research Centre, University of Warsaw, Żwirki i Wigury 101, 02089 Warsaw, Poland; ddziubak@chem.uw.edu.pl (D.D.); slasek@chem.uw.edu.pl (S.S.)

**Keywords:** lipid rafts, Langmuir–Blodgett, electrochemical impedance spectroscopy, atomic force microscopy

## Abstract

A model biomimetic system for the study of protein reconstitution or drug interactions should include lipid rafts in the mixed lipid monolayer, since they are usually the domains embedding membrane proteins and peptides. Four model lipid films composed of three components: 1,2-dioleoyl-sn-glycero-3-phosphocholine (DOPC), cholesterol (Chol) and sphingomyelin (SM) mixed in different molar ratios were proposed and investigated using surface pressure measurements and thermodynamic analysis of the monolayers at the air–water interface and imaged by Brewster angle microscopy. The ternary monolayers were transferred from the air–water onto the gold electrodes to form bilayer films and were studied for the first time by electrochemical methods: alternative current voltammetry and electrochemical impedance spectroscopy and imaged by atomic force microscopy. In excess of DOPC, the ternary systems remained too liquid for the raft region to be stable, while in the excess of cholesterol the layers were too solid. The layers with SM in excess lead to the formation of Chol:SM complexes but the amount of the fluid matrix was very low. The equimolar content of the three components lead to the formation of a stable and well-organized assembly with well-developed raft microdomains of larger thickness, surrounded by the more fluid part of the bilayer. The latter is proposed as a convenient raft model membrane for further physicochemical studies of interactions with drugs or pollutants or incorporation of membrane proteins.

## 1. Introduction

Lipid rafts [1,2] are microdomains present in the structure of eukaryotic biological membranes [3]. They are an inherent component of the biological membranes and are responsible for their properties and proper functioning. Microdomains are involved in many cell functions such as membrane transport and signal transduction. These structures are found not only in the cell membranes but also in the membranes of the mitochondria and the endoplasmic reticulum (ER) [4,5,6]. Although they are very dynamic structures that frequently change their positions [7,8], they are also regions of anchoring for many proteins [9]. Lipid rafts are mainly composed of sphingolipids and sterols, which results in the different packing and thickness of the lipid layer as compared to the remaining part of the membrane [5,10].

**Figure 1:**
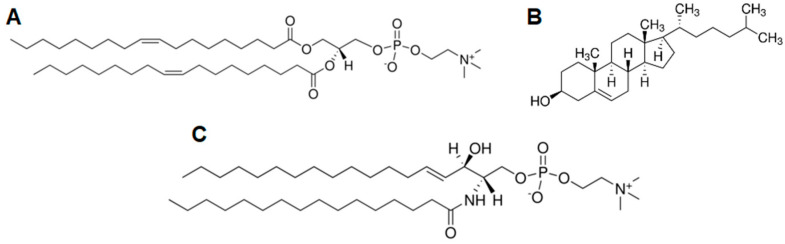
Chemical structures of lipids: (**A**) 1,2-dioleoyl-sn-glycero-3-phosphocholine (DOPC); (**B**) Cholesterol (Chol); (**C**) Sphingomyelin (SM).

Sphingomyelin (Figure 1) is a common component of biological membranes, not only manifested in lipid rafts [10]. It can be metabolized to ceramides, thus being a precursor to signaling lipids [11,12]. Sphingomyelin interacts specifically with cholesterol molecules (Figure 1) [13,14,15,16,17]. Both form a liquid-ordered (*L_o_*) structure [18,19], separated from the liquid disordered (*L_d_*) structure mainly composed of glycolipids [18,20]. In lipid rafts, cholesterol (Chol) and sphingomyelin (SM) molecules interact through hydrogen bonds, mainly between the hydroxyl group of cholesterol and the amide group of sphingosine, but also through hydrophobic interactions between rigid sterol rings and sphingomyelin acyl chains [21,22]. In contrast, phospholipids containing choline polar heads are present in raft structure to balance the high level of lipids leading to layer order. The chain length of fatty acid residues and their degree of unsaturation are other factors determining the quality of lipid rafts [23,24,25]. Choline lipids such as 1,2-dioleoyl-sn-glycero-3-phosphocholine (DOPC) are common biological membrane-forming lipids and their interaction with cholesterol results in a better packing of lipids in the layer, which distinguishes the rafts from the rest of the membrane [17,24,26,27,28,29]. Cholesterol is mostly located in the hydrophobic part of the layer, just behind the hydrophilic polar heads of phospholipids [23,30,31,32,33,34], but it also interacts through hydrogen bonds with the ester groups of DOPC molecules [23]. On the other hand, DOPC and sphingomyelin also interact through the polar heads, which allows cholesterol molecules to locate between hydrophobic lipid chains, filling the voids between them [35].

The simplest models of rafts consist of two components only: cholesterol and sphingomyelin [28,35,36]. Such models do not provide, however, the fluidity of the matrix, which accommodates the rafts. Therefore, systems containing phosphatidylcholines are also studied. The exact type of PC lipid used is a contentious issue. The most common ones include POPC [20,36,37,38], DPPC [16,32] and DOPC [26,27,29] and meet the condition of *L_o_/L_d_* phase separation [29,30,39]. Model systems with an equimolar ratio of the components—the PC:Chol:SM 1:1:1 model [27], or ternary models with an increased amount of cholesterol or an increased amount of DOPC were proposed [26,40]. The latter provides a more liquid structure of the membrane. These different models of lipid raft membranes have not been compared so far in terms of their surface and electrical properties.

In this work, we characterize four models of lipid rafts differing in the molar ratio of the three main components: DOPC, cholesterol, and sphingomyelin. The ternary monolayers are formed at the air–water interface and their surface properties are described using the Langmuir technique including the analysis of thermodynamic functions and are supplemented with Brewster angle microscopy (BAM) imaging. The combination of Langmuir–Blodgett and Langmuir–Schaefer techniques allowed for the preparation of supported model bilayers with an architecture resembling the natural membranes. The application of electrochemical techniques such as alternative current voltammetry (ACV) and electrochemical impedance spectroscopy (EIS) provided information on the electrical properties as well as organization and homogeneity of the supported model rafts depending on their exact molar content. Another way to visualize the lipid domains corresponding to lipid rafts [19,41] is atomic force microscopy (AFM) [42]. This combined approach enabled the characterization and comparison of different models of lipid membranes with respect to their surface and electrochemical properties. It allowed us also to answer a frequently asked question [43,44]: what is the significance of individual lipids in the lipid rafts?

## 2. Results and Discussion

### 2.1. Langmuir Monolayer Studies

**Figure 2:**
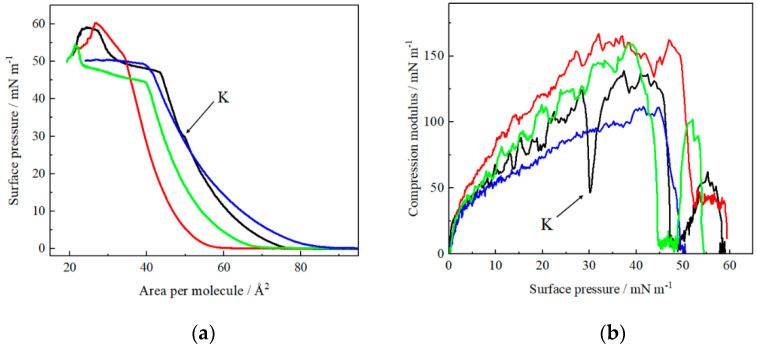
Surface pressure-area per molecule (π-A) isotherms of (**a**) DOPC:Chol:SM 1:1:2 (green), DOPC:Chol:SM 1:2:1 (red), DOPC:Chol:SM 2:1:1 (blue) and DOPC:Chol:SM 1:1:1 (black) monolayers formed on PBS buffer and (**b**) compression modulus vs. surface pressure plot. (T = 21 ± 1 °C).

The presented four models of lipid rafts differ in the lipid molar ratio of the three components. The surface properties of such models were first investigated using the Langmuir technique (Figure 2 and Table S2).

Each component of the ternary model has a large impact on its structure and thus on its surface properties (Figure S1 and Table S1), which is indicated by the high variability in the course of the *π-A* isotherms. In the first model, the amount of sphingomyelin in the layer was increased, which manifested in the largest changes in the structure in the range of high surface pressure values, leading to a partial collapse. An interesting phenomenon is a significant decrease in the value of *A*_0_ before and after the partial collapse, where *A*_0_ is equal to 52.7 Å^2^ and *A*_0_ after partial collapse is 31.0 Å^2^ (Figure 2a, green and Table S2). The maximum *C_s_^−^*^1^ value (~162 mN/m) is practically the same as for the model with the increased amount of cholesterol, which is not surprising, since both components (Chol and SM) form rigid structures at the air–water interface (Figure S1). Additionally, the Chol:SM 1:2 ratio is particularly supposed to produce the strongest attractive interactions between the two lipids leading to the formation of highly stable ‘surface complexes’ [45], which can form more tightly packed structures, such as lipid rafts. They are based on the favorable geometric packing of SM and Chol molecules due to their complimentary critical packing parameters equal to 1.22 and 0.57 indicating their inverted conical shape and truncated conical shape, respectively. Raman microscopy measurements [46] are the additional evidence of the presence of SM in the tightly packed lipid.

**Figure 3:**
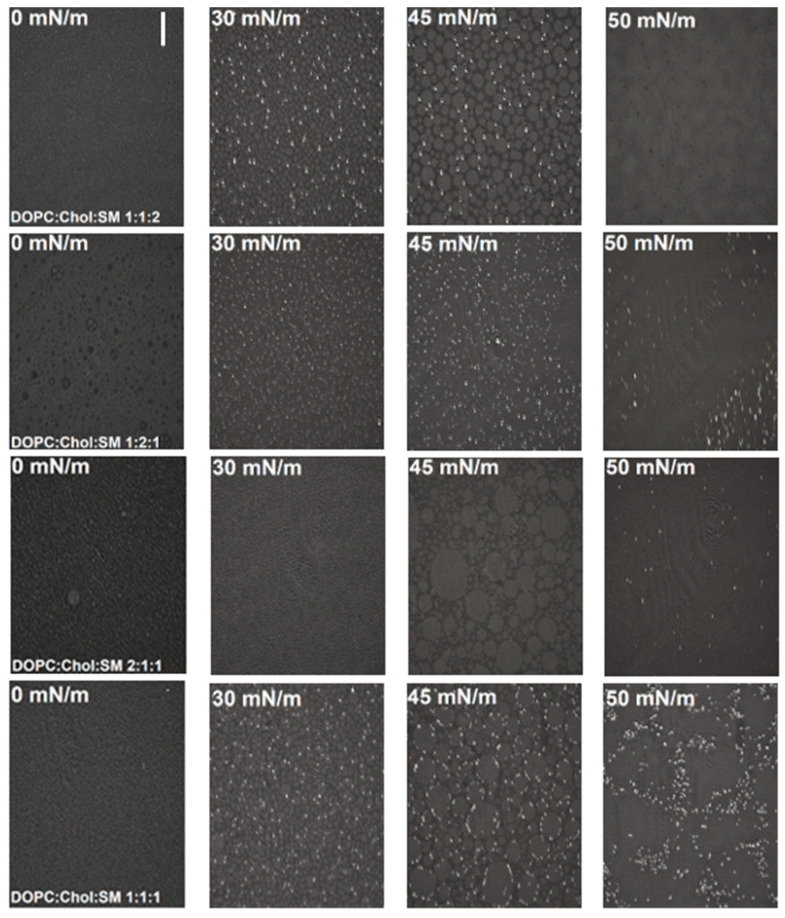
BAM images obtained at selected surface pressures for monolayers of model lipid rafts formed on PBS buffer pH 7.4 (T = 21 ± 1 °C). The scale bar is 100 µm.

A change in the structure of DOPC:Chol:SM 1:1:2 taking place at approximately 45 mN/m leads to the fluidization of the layer, indicating complete removal of the rafts from the layer. This conclusion is confirmed by the BAM images (Figure 3), where during compression the white raft aggregates are visible until reorganization, and at 50 mN/m a complete homogeneity of the monolayer is visible. The formation of aggregates indicates a separation of the *L_o_/L_d_* phases, which is in line with the literature reports [47,48], where the separation of the Chol-SM structures from the liquid disordered part of the layer is explained (see also [49] and phase diagram of the ternary mixture DOPC/PSM/Chol).

In the case of the monolayers with an increased amount of cholesterol (DOPC:Chol:SM 1:2:1) the LC phase (*C_s_^−^*^1^*_max_* = 169 mN/m) was observed. The shape of the *π*-*A* isotherm (Figure 2/red) reflects the influence of cholesterol on the ternary assembly. The isotherm is strongly shifted towards the isotherm corresponding to the formation of single-component cholesterol monolayer (Figure S1) [50]. At π > 30 mN/m both isotherms overlap. The *A*_0_ for this well-organized ternary monolayer is relatively low (48.1 Å^2^) (Table S2), which confirms the strong influence of cholesterol on the assembly. Monolayers containing excess of cholesterol are characterized by more dense packing, which is consistent with the phase diagrams [51]. The structural reorganization at higher surface pressure is also observed but it is not as clearly resolved as for DOPC:Chol:SM 1:1:2 and DOPC:Chol:SM 1:1:1 cases, probably due to the large effect of cholesterol—only the excess of this component can be pushed out of the monolayer (Figure 2b/red). The value of *C_s_^−^*^1^ does not drop to zero at the minimum at approximately 50 mN/m, which indicates that there is no collapse [52]. The break point in the isotherm, at which the reorganization begins, corresponds exactly to the point at which the collapse of the DOPC monolayer starts (Table S1). Due to the fairly strong packing of lipids, this ternary layer is also distinguished by high stability (*π* = 59.5 mN/m). BAM images in the very beginning of compression indicate the coexistence of gas and liquid phases (Figure 3). At around 30 mN/m, the effect of cholesterol becomes apparent through the appearance and thickening of the structures of fine white ovules. However, at 45 mN/m the domains are not as characteristic as in the case of the DOPC:Chol:SM 1:1:2 and/or DOPC:Chol:SM 1:1:1 monolayers and the white ovules are randomly distributed. Further compression leads to the formation of a more homogeneous layer. At the surface pressure value of 50 mN/m only a few white, very small structures remain, which rather indicates lipid aggregation. Therefore, based on the isotherm data and BAM images it may be suggested that the formation of lipid rafts is not favored for this model.

The next lipid raft model consists of the DOPC:Chol:SM 2:1:1 molar ratio (Figure 2/blue). The value of the surface pressure of the collapse corresponding to this monolayer is 49.4 mN/m (Table S2). It is also not surprising that the *A*_0_ for the monolayer with the increased amount of DOPC is greater than in the other cases and is equal to 62.0 Å^2^, since it reflects the relatively high value of the main component—DOPC. Also, it is characterized by a more liquid nature, where it fits the LE-LC phase through the *C_s_^−^*^1^*_max_* = 98 mN/m [26,53]. The maximum of the compressibility coefficient falls into high values of the surface pressure, i.e., about 45 mN/m which is reflected in the BAM images (Figure 3). They confirm the more liquid nature of the monolayer in relation to other mixtures, which is in line with the work of Maté et al. [53]. From the very beginning, characteristic fine structures and the coexistence of phases are observed. In this case, until the surface pressure of 40 mN/m no particular changes in the layer morphology are observed. At this point, large circular domains with very rare white particles appear [42]. However, there are no white, bright raft structures observed for the other models (Figure 3). As a result, the monolayer is relatively homogeneous throughout the compression cycle, even at the surface pressure of 50 mN/m.

An in-depth analysis of the DOPC:Chol:SM (Figure 2/black) lipid raft model of equal molar ratio of components was performed. An interesting phenomenon that appears only with the equimolar mimetic model is the kink occurring at a surface pressure of 30 mN/m. This type of isotherm bending indicates changes in the molecular structure of the monolayer at the air–water interface. In this case, it may indicate the moment of lipid raft formation, since the surface pressure, at which it occurs, corresponds to the surface pressure, at which the monolayer’s surface properties and organization starts to resemble those typical for real bilayers forming biological membranes [54]. Interestingly, such a change in the structure observed as a kink on the isotherm occurs in lipid biomimetic models, where one of the components is cholesterol [55,56], which is the main raft-forming individual [5,57,58,59]. The range of the surface pressures from 30 to 35 mN/m, corresponds to conditions met in real biological membranes [54,59,60,61]. Another noticeable change in the course of the isotherm occurs in the range of high surface pressure values, which in this case indicates a partial collapse (*π_coll/_*_1_ = 47.8 mN/m), reflected in the minimum reaching zero on the *C_s_^−^*^1^-*π* plot (Figure 2b). It could mean the breakdown of the two-dimensional structure of the layer by removing components from it. Alternatively, it may indicate the displacement of lipid rafts from the ternary layers [21,22], which also is in line with phase diagram [13]. A further increase in the isotherm is due to the presence of residual sphingomyelin molecules, as indicated by the same value of the area after the partial collapse (*A*_0_ after partial collapse = 50.5 mN/m) and for a single-component monolayer of SM (*A*_0_ = 50.7 Å^2^, Table S1) [62]. Due to the maximum value of the reciprocal of compression modulus equal to 125 mN/m, it can be determined that the interactions between the molecules (DOPC-Chol-SM) are quite strong, and the layer can be assigned to the liquid condensed (LC) phase. Brewster angle microscopy confirmed the LC nature of this layer (Figure 3). However, BAM images for the DOPC:Chol:SM 1:1:1 monolayer show the immediate appearance of small oval-shaped structures even at low surface pressures—the coexistence of the gas and liquid phases is visible even at π equal to 0 mN/m. With further compression, the bright-gray structures are denser, with white ovules at the edges indicating the formation of lipid raft domains (composed mainly of Chol molecules). At the surface pressure of 50 mN/m there is a change in the morphology of the structure, where raft aggregates were forced out above the layer surface and a more homogeneous layer is observed in between the aggregates.

**Figure 4:**
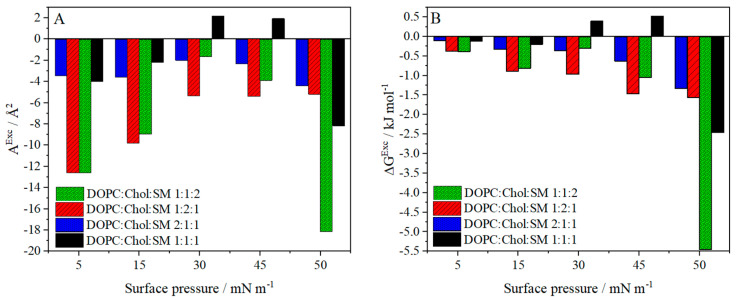
Values of (**A**) the excess area (∆*A*^*Exc*^) and (**B**) the excess free energy (∆*G*^*Exc*^) calculated at selected surface pressures for DOPC:Chol:SM 1:1:2 (green), DOPC:Chol:SM 1:2:1 (red), DOPC:Chol:SM 2:1:1 (blue) and DOPC:Chol:SM 1:1:1 (black) monolayers.

An additional way to assess the quality of the multi-component layer and the nature of the interactions between the components is to calculate the excess parameters—the excess area (*A^Exc^*, Equation (3)) and the Gibbs free energy (∆*G^Exc^*, Equation (4)) [63]. The summary of the calculated values in relation to the surface pressure is shown in Figure 4.

In the case of the three non-equimolar models, the values of both excess area and excess free energy are negative for all surface pressures (Figure 4). The observed decrease in the ∆*G^Exc^* with the increasing surface pressure implies that the ternary systems become more thermodynamically stable. For the DOPC:Chol:SM 1:2:1 and DOPC:Chol:SM 1:1:2 monolayer, these values are more negative than for the DOPC:Chol:SM 2:1:1 monolayer. The miscibility of the components in the ternary layers depends on the cholesterol content, as reported by Stottrup et al. [51]. Its low content causes the so-called α-regions, while high cholesterol content creates β-regions (both characterized by immiscibility according to phase diagrams). Therefore, it may be supposed that for the two former models the interactions are more attractive than for the monolayer with the prevailing amount of DOPC. The analysis of the Gibbs free energy at the surface pressure of 30 mN/m shows that the most stable system is the one with the increased amount of cholesterol (∆*G^Exc^* value ~1000 J/mol). For surface pressure value equal to 30 mN/m, the percent condensation of the components in the layers (*%A*) was also calculated (Equation (5)). It was equal to 3.6%, 3.9% and 11.9% for DOPC:Chol:SM 1:1:2, DOPC:Chol:SM 1:2:1, and DOPC:Chol:SM 2:1:1, respectively. The high *%A* value for a monolayer with an increased amount of cholesterol indicates a stronger condensation of the layer [47], which correlates with the *π-A* isotherm measurements, where the packing degree is greatest (*A*_0_, Table S2).

The equimolar ternary monolayer exhibits different behavior than the other three model systems. Only in this case, positive deviations from the ideal state were recorded occurring in the range of surface pressure from 30 mN/m to 45 mN/m. This range covers the layer compression from the moment of the probable formation of the raft (kink on the *π-A* isotherm, Figure 1B/black) to the moment of a partial collapse. It also corresponds to the biologically relevant surface pressures [54]. *A^Exc^* > 0 indicates the presence of more repulsive or less attractive forces between the molecules within the ternary layer compared to individual components, which is additionally reflected in the BAM images (Figure 3), where separation of domains in the layer is visible. A negative value of %condensation at 30 mN/m resulting from the additivity rule for an equimolar mixture indicates the separation of components in the layer due to the occurrence of raft domains. All these observations confirm the formation of the unstable structures such as lipids rafts within the model DOPC:Chol:SM 1:1:1 monolayer explained as due to the lack of conformability between the rigid structure of cholesterol and the cis double bond (C9:C10) in the DOPC molecule [63]. For comparison, for the DOPC:Chol or Chol:SM two-component monolayers, the excess free energy values are negative [14,44]. It indicates that three components mixed in an appropriate stoichiometry lead to the formation of lipid rafts with reduced thermodynamic stability of the mixed layer.

**Figure 5:**
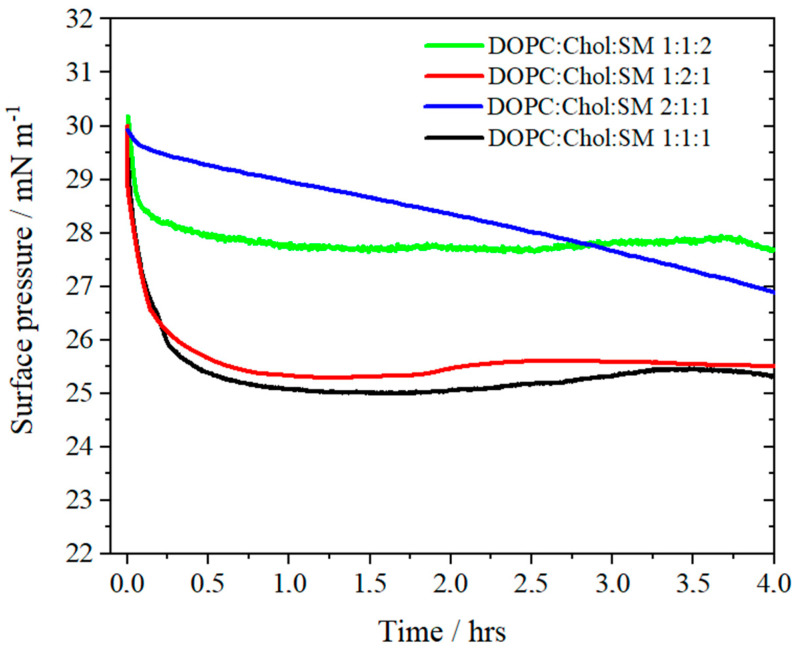
The surface pressure vs. time plots for DOPC:Chol:SM 1:1:2 (green), DOPC:Chol:SM 1:2:1 (red), DOPC:Chol:SM 2:1:1 (blue) and DOPC:Chol:SM 1:1:1 (black) Langmuir monolayers compressed to 30 mN/m on PBS buffer, pH 7.4 (T = 21 ± 1 °C).

The purpose of registering the surface pressure changes for over four hours (Figure 5) was to assess the stability of the film in time. The three remaining model membranes stabilize within half an hour. For the DOPC:Chol:SM 1:1:2 and DOPC:Chol:SM 1:2:1 monolayers, the plateau is obtained at 28 mN/m and 26 mN/m, respectively. It turns out that the most liquid layer with the predominant molar amount of DOPC is most unstable, as evidenced by the continuous decrease in surface pressure in time, in contrast to the equimolar model, or that with a predominance of cholesterol, which is stable and therefore can be effectively transferred onto solid supports. The failure to reach the surface pressure plateau in the monolayer where the unsaturated lipid predominates (DOPC:Chol:SM 2:1:1) can be explained by the oxidation of the lipid double bonds as suggested by Qiao et al. [64], who used an argon chamber to decrease the contribution of this process.

### 2.2. AC Voltammetry

**Figure 6:**
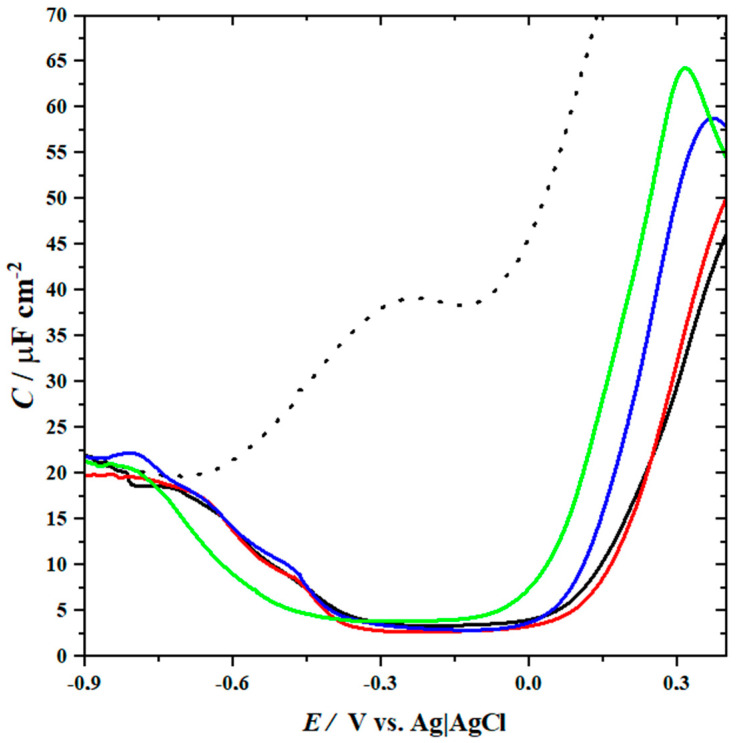
AC voltammetry measurements of the Au (111) electrode with the supported DOPC:Chol:SM 1:1:2 (solid green), DOPC:Chol:SM 1:2:1 (solid red), DOPC:Chol:SM 2:1:1 (solid blue), DOPC:Chol:SM 1:1:1 (solid black) bilayer and the bare gold electrode (dashed black).

The electrode modified with lipid bilayer can be considered as a capacitor. By analyzing the differential capacitance versus applied potential (Figure 6), the potential window, at which biomimetic membranes are most stable and densely packed, can be determined [65]. At positive potentials, the capacity is rather high, which indicates poorly organized lipids in the layer [66]. Shifting the potential to more negative values causes membrane reorganization. As a result, a well-packed bilayer is formed. It is reflected in the sharp drop of capacitance at potential ~0.0 V vs. Ag|AgCl. In the range of potential from 0.0 V to −0.3 V vs. Ag|AgCl, the value of capacitance does not vary noticeably and reaches the minimum for all the investigated four models of lipid rafts. However, for the DOPC:Chol:SM 1:1:2 bilayer, a slight shift of the minimum of capacitance towards more negative potentials is observed, which may be attributed to the formation of the specific surface complexes of Chol:SM mixed in the 1:2 molar ratio [45]. The formation of these complexes changes the charge distribution, as can be seen in the charge density vs. potential plot (Figure S2). For DOPC:Chol:SM 1:1:2 bilayer, the determined potential of zero free charge (*E_pzfc_*) [67,68] is +270 ± 10 mV, while for the DOPC:Chol:SM 1:1:1, DOPC:Chol:SM 1:2:1 and DOPC:Chol:SM 2:1:1 films it is: +330 ± 20, +290 ± 20 and +300 ± 10 mV, respectively (Table S3). The values of the minimum capacitances are similar, in the range of 3.5 to 2.6 µF cm^−2^ (Table S3). Since an ideal defect-free lipid bilayer has the capacitance of ca. 0.8 µF cm^−2^ [69], it is evident that all the investigated model membranes contain some defects and do not form compact homogenous layers. At the minimum of capacitance, where the membrane is the most densely packed, the coverage of the Au (111) surface can be calculated to be in the range of 92–95% [66]. During further negative polarization, an increase in capacitance is observed. This behavior can be ascribed to electroporation. A lipid bilayer lifts from the surface of the electrode, while water accumulates in the region between the membrane and the solid substrate [70,71]. At most negative potentials it finally leads to a complete detachment of the membrane from a solid substrate, which is manifested by the overlapping of the curve with that obtained for a film-free Au (111) substrate (Figure 6). For the DOPC:Chol:SM 1:1:2 model membrane, the detachment occurs at the most negative potential showing that the complexes present in such a film may adsorb more strongly at the gold surface.

### 2.3. Electrochemical Impedance Spectroscopy

**Figure 7:**
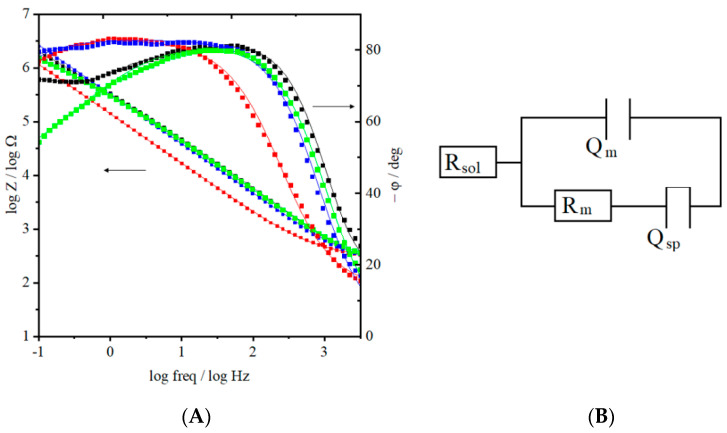
(**A**) Bode plots presenting logarithm of impedance and phase angle as a function of the frequency for proper bilayers at potential −0.2 V vs. Ag|AgCl. Dots represent the measured spectra, while lines represent data obtained by fitting spectra to an equivalent circuit. Spectra obtained for DOPC:Chol:SM 1:1:2 (green), DOPC:Chol:SM 1:2:1 (red), DOPC:Chol:SM 2:1:1 (blue), DOPC:Chol:SM 1:1:1 (black) and (**B**) Equivalent circuit, used to fit obtained data, R_sol_ and R_m_ represent the resistance of solution and membrane, respectively, while Qm and Qsp are constant phase element of membrane and spacer between the bilayer and Au (111) surface.

According to the data obtained from ACV measurements, the minimum capacitance obtained for each model raft membrane is at potential ~−0.20 V vs. Ag|AgCl (Figure 7). Therefore, this potential was chosen to perform the EIS experiments and to obtain the impedance spectra. Figure 7A presents the recorded spectra and fitted equivalent circuit shown in Figure 7B. The selected circuit assumed that there is a thin layer of water between the membrane and the electrode [72]. The correlation between the obtained and the fitted data is high, which proves that the equivalent circuit was chosen correctly. Two of the four lipid raft models are similar in lipid packing, according to the obtained impedance spectra: with the highest molar ratio of DOPC (DOPC:Chol:SM 2:1:1) and Chol (DOPC:Chol:SM 1:2:1). The phase angle in a frequency region lower than 100 Hz reaches a plateau and is almost −90°. This behavior is characteristic of capacitive elements. In this case, it would indicate a well-organized and homogenous bilayer. It is supported by the BAM images, which show for these two model membranes the most homogeneous monolayers at the surface pressure of 30 mN/m, at which the layers were transferred (Figure 3). These systems also are characterized by the highest value of resistance (Table 1). However, the model with the highest concentration of Chol (DOPC:Chol:SM 1:2:1) is less permeable. The relation between the resistance of the membrane and cholesterol content was described previously [73] and explains this fact. The presence of cholesterol not only reduces the mobility of lipids in the membrane leading to an increased stiffness but also seals it, which is manifested by its lower water permeability. It is also supported by the high *%A* value obtained from thermodynamic calculations showing high packing degree (*A*_0_, Table S2 and Figure 4). Moreover, both models are characterized by the highest value of resistance, which confirms the most densely packed structure of lipids (Table 1). The capacitance of the membrane is also comparable, which might be related to the similarities of these membranes.

Different behavior is observed for a model with the highest molar ratio of SM (DOPC:Chol:SM 1:1:2). On the phase angle plot the increase of phase angle at low-frequency value is observed (Figure 7A). Moreover, no plateau is noticeable. The shape of the curve showed a mixed nature of the layer. According to the available results, we can conclude that this system is characterized by the lowest homogeneity, which is also proved by BAM images. The bilayer deposited on the surface of the electrode is fluid, and lipids do not form densely packed structures. This is directly reflected by the resistance of the membrane (Table 1) and agrees with the results obtained from ACV measurements, where this model membrane shows the largest value of capacitance. The obtained value of Q_sp_, which is the highest for this model, suggests a large contribution to the accumulation of water in the submembrane region. This reflects the non-homogeneous structure of the lipid system in the membrane and the fluidity; therefore, it shows high permeability.

For the model with equimolar lipid composition (DOPC:Chol:SM 1:1:1), a shallow minimum is noticeable on the phase angle plot (Figure 7A). Such a shape might indicate two independent domains with different mass transport properties. In other words, this layer has defects and is characterized by increased roughness. We can consider it as a layer with typical rafts. These small sub-micrometer range domains vary in thickness from the rest of the bilayer. As a result, unevenness with differences in the thickness (1–2 nm) of the resistance layer is formed [74], where thicker parts come from rafts. The permeability of this system is indirectly compared to the previous models discussed.

### 2.4. Atomic Force Microscopy

**Figure 8:**
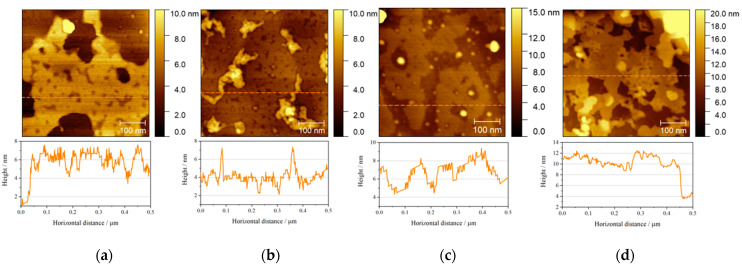
Upper panel: AFM images (500 × 500 nm^2^) of lipid rafts models (**a**) DOPC:Chol:SM 1:1:2, (**b**) DOPC:Chol:SM 1:2:1, (**c**) DOPC:Chol:SM 2:1:1 and (**d**) DOPC:Chol:SM 1:1:1; Lower panel: vertical profiles corresponding to orange dashed lines in the above AFM images.

**Figure 9:**
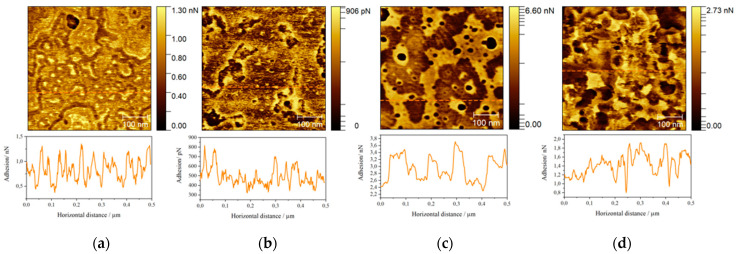
Upper panel: adhesion maps (500 × 500 nm^2^) for (**a**) DOPC:Chol:SM 1:1:2, (**b**) DOPC:Chol:SM 1:2:1, (**c**) DOPC:Chol:SM 2:1:1 and (**d**) DOPC:Chol:SM 1:1:1 layers; Lower panel: vertical profiles corresponding to orange dashed lines in the above AFM images.

To verify the structural differences of four mimetic models of lipid rafts we have used AFM imaging. This technique is suitable for assessing the morphology of the lipid layer [75,76,77] due to its subnanometer resolution along *z*-axis. Nevertheless, Yuan et al. [41] pointed out that visualization of lipid rafts may encounter some difficulties, as what is observed may result from differences in packing density. The figures below show the differences in height between the domains in the lipid layers and the cross-sectional profiles (Figure 8), while the Figure 9 shows the distribution of the adhesive forces corresponding to the scanned areas.

Figure 8a shows the surface of the gold electrode covered with a DOPC:Chol:SM 1:1:2 layer, where mainly two areas can be distinguished [27]. The presence of darker patches indicates a homogeneous structure (height ~ 2 nm), which is more fluid and viscous. The central, bright region in this image is characterized by a tighter lipid packing. Owing to the measurement of the adhesive forces (Figure 9), it can be shown that this region represents a stiffer domain. This pattern may be explained by the formation of the tight Chol:SM 1:2 complexes [45]. It is a large region, laterally reaching around 400–500 nm [78], protruding above the liquid structure by about 4 nm. A characteristic feature of this layer is the presence of defects in the rigid structure, which Sakamoto et al. describe as resulting from the sphingomyelin in the layer [27,79]. The characteristic “pin-holes” in this case reach depths of ~1.7 nm.

The cholesterol-enriched system tends to have two phases (Figure 8b). The occurrence of inhomogeneous aggregates and/or crystallites is observed here, which is related to cholesterol [79,80]. The size of the aggregates ranges from a few to several dozen nanometers. However, it is shown that the rigid domains are much finer than in the other cases [78]. In this model, a large disorder and building-up of layers on the bilayer matrix with a thickness of approx. 4 nm is observed. It should also be noted that the darker areas are not homogeneous, and there are cavities with a depth of approx. 2 nm.

As predicted from previous techniques, the DOPC:Chol:SM 2:1:1 layer (Figure 8c) is the most liquid of all the models discussed. Moreover, the separation of ordered (*L_o_*, lighter patches) and disordered (*L_d_*, darker patches) phases is visible [8,53]. It should be noted that the thickness of liquid lipid bilayers containing DOPC depends on their degree of hydration [81], but in general DOPC-based bilayers are considered homogeneous [82]. Here, it is possible to indicate the existence of lipid rafts with the lateral size from 100 to 200 nm [19], and their height relative to the bilayer is about 1–2 nm, which is consistent with the dimensions of lipid rafts isolated from real cell membranes [42].

DOPC:Chol:SM 1:1:1 is a system that combines the features of all the models discussed above. This structure is based on a bilayer (thickness of ~4 nm), with multilayers built up on top of it. It may be caused by high asymmetry of the system, which promotes aggregation and the formation of multilayers upon transfer [79]. The differences in height reach even 20 nm, which means that there are at least four bilayers. Nevertheless, AFM images also show more flattened areas, where lipid rafts, similar to those for the DOPC:Chol:SM 2:1:1 model, can be found. Moreover, pin-holes characteristic for sphingomyelin layers and aggregates/crystallites present in cholesterol-enriched layers are also visible in this model.

The adhesion measurements (Figure 9) show the same trends in each case. More liquid domains exhibit greater adhesion than the more ordered ones. This observation is in line with the data reported in the literature, since adhesion force depends on the physical state of the membrane. As demonstrated by Leonenko group [83], the transition of the lipid bilayer from the gel to liquid state leads to a gradual increase in the adhesion force. The smallest differences in forces are visible for the DOPC:Chol:SM 1:1:2 and 1:2:1 models, i.e., those that are characterized by the tightest lipid packing. The largest phase separation was observed for the most liquid of the discussed layers, as in the study by Maté et al. [53]. The multilayering of the equimolar model is also manifested in the adhesion measurements, where the areas of more or less liquid as well as rigid domains are observed. In the case of this work, we are dealing with the coexistence of different domains, where the membrane occurs in different physical states reflected in the differences in adhesion.

## 3. Materials and Methods

### 3.1. Materials

Three high purity (≥99%) lipids characteristic for lipid rafts were used. Cholesterol (Chol) was purchased from Sigma-Aldrich (Darmstadt, Germany). Both 1,2-dioleoyl-sn-glycero-3-phosphocholine (DOPC) and egg sphingomyelin (SM) were obtained from Avanti Polar Lipids (Alabaster, AL, USA). Solvents, anhydrous chloroform, and methanol (Sigma-Aldrich, Darmstadt, Germany) were used to prepare lipid stock solutions, all at a final concentration of about 0.3 mg/mL. Chol and DOPC were dissolved in chloroform and to improve the solubility of SM solution it was prepared in a mixture of chloroform:methanol 4:1 *v*/*v*. Four different ternary solutions were prepared by mixing the lipids to obtain the solutions with the different final molar ratios: DOPC:Chol:SM 2:1:1, DOPC:Chol:SM 1:2:1, DOPC:Chol:SM 1:1:2, and DOPC:Chol:SM 1:1:1. The biomimetic layers were formed on the phosphate-buffer saline (PBS) in MilliQ water (18.2 MΩ·cm) as a subphase with the concentration of 0.01 M and pH of 7.4. Such conditions correspond to the physiological conditions in the intercellular environment.

### 3.2. Methods

#### 3.2.1. Langmuir Technique

KSV Instrument (Finland) containing a hydrophobic Langmuir trough was used to perform the surface pressure (π)-area per molecule (*A*) isotherms. The measuring set was also equipped with hydrophilic barriers and a surface pressure sensor—Wilhelmy balance with Wilhelmy plate (filter paper). The entire system was controlled by KSV software (KSV 5000, KSV-Nima, Espoo, Finland). The experiment began with the preparation of the subphase. After pouring the PBS solution into a Langmuir trough (with a total area of 243 cm^2^), its surface was thoroughly cleaned. Using a Hamilton microsyringe (±1.0 μL), the lipid solution was spread on the surface of the subphase, and the volatile solvent was allowed to evaporate. Then, the symmetric compression of the barriers at the rate of 10 mm/min was performed. Simultaneously, the changes in the surface pressure were recorded during the formation of a monolayer by lipids. Measurements were carried out under the laboratory conditions (21 ± 1 °C).

To compare the properties of the investigated layers apart from the area per molecule in the well-organized layer (*A*_0_) obtained by the extrapolation of the isotherm in its steepest part to the zero surface pressure and area at 30 mN/m (*A_π_* = 30 mN/m), the area per molecule and the surface pressure of the two collapses (before and after the structural change) were also determined. An important aspect in comparing lipid rafts models is the assessment of their elastic properties, which can be estimated based on the reciprocal of compression modulus (*C_s_*^−1^) [84]:

(1) $$C_{s}^{-1}=-A{\left(\frac{d\pi }{dA}\right)}_{T}$$

In this formula, *π* is the surface pressure and A is the area per molecule, both parameters are obtained from isotherm data. The maximum value of *C_s_*^−1^ shows the elastic properties of the layer and the arrangement of particles in relation to each other, accurately described in the literature [85,86]. *C_s_*^−1^ values between 0 and 12.5 mN/m correspond to a gas phase (G), when molecules are freely occupying the interface and do not interact with each other. The values between 50 mN/m and 100 mN/m correspond to the liquid-expanded phase (LE), while the values in range from 100 mN/m to 250 mN/m—to liquid-condensed (LC) phase. C_s_^−1^ exceeding 250 mN/m are characteristic for very tightly packed molecules in the layer interacting with each other through both polar heads and hydrophobic tails leading to the formation of a solid monolayer (S).

Moreover, thanks to the Langmuir technique, the miscibility of the components for the multi-component layer and the type of forces responsible for the interactions between the molecules can be determined. The positive values of excess area (*A^Exc^*, Equation (3)) [14] indicate repulsive or weakly attracting interactions between the components, which indicates immiscibility. When *A^Exc^* value is negative, the interactions are attractive or weakly repulsive, which indicates the miscibility of the components.

(2) $$A_{1\ldots N}^{id}=\sum_{1}^{N}A_{i}X_{i}$$

(3) $$A^{Exc}=A_{1\ldots N}-A_{1\ldots N}^{id}$$

(4) $$\Delta G^{Exc}=N_{A}\int_{0}^{\pi }A^{Exc}d\pi$$

(5) $$\%A=\frac{A_{1\ldots N}^{id}-A_{1\ldots N}}{A_{1\ldots N}^{id}}\cdot 100\%$$

In the formulas given above, *A_i_* and *X_i_* are the area per molecule of the single component and mole fraction of the component in the mixture, respectively. This allows for the calculation of the average value of the area occupied by a single component in an ideally miscible multicomponent layer ($A_{1\ldots N}^{id}$, Equation (2)). The excess area can be calculated taking into account $A_{1\ldots N}$, which is the area read from the isotherm for mixed monolayers at a given surface pressure. By using $A_{1\ldots N}$ and $A_{1\ldots N}^{id}$, the percent condensation of a monolayer (%*A*, Equation (5)) at a given surface pressure can be determined [47]. Additionally, using the excess area, the Gibbs free energy (∆*G^Exc^*, Equation (4)) [14] can be calculated, explaining the thermodynamic stability of the layer. For ideal Langmuir films, both parameters (*A^Exc^*, ∆*G^Exc^*) are equal to 0. Any deviation from this value indicates the presence of interactions other than those present in the monolayers of individual components.

Using the same laboratory conditions as in the case of recording isotherms, the changes in the surface pressure of the layer over time (π-t) were recorded. After the layer was compressed to 30 mN/m, the barriers remained motionless, and the surface pressure changes were measured for about 4 h. During this time, it was possible to determine the stability of the structures corresponding to the biomimetic lipid rafts.

#### 3.2.2. Brewster Angle Microscopy (BAM)

Brewster angle microscopy (BAM) was used to follow the changes in the morphology and properties of Langmuir monolayers. A trough with an area of 841 cm^2^ was used for BAM measurements. The method of preparing the experiment was the same as the measurement of *π-A* isotherm. The BAM images correspond to an area of 800 μm × 430 μm and were captured with the 2 µm resolution.

#### 3.2.3. Bilayer Transfer onto a Solid Support

The Langmuir–Blodgett (LB)—Langmuir–Schaefer (LS) method was used to reproduce the biomembrane architecture on the solid support—Au (111) electrode (MaTeck Material-Technologie & Kristalle GmbH, Jülich, Germany). The electrodes required prior cleaning and were therefore thoroughly washed in piranha solution (H_2_SO_4_/H_2_O_2_, 3:1 *v*/*v*), rinsed with copious amounts of MilliQ water and then flame-annealed, which also provided hydrophilic properties of the solid substrate [87]. The Arrandee electrodes (11 mm × 11 mm) with gold evaporated onto borosilicate glass were used as supporting substrates for the bilayers in AFM experiments. The method of their preparation was the same as described above.

The two-stage process of obtaining the bilayers begins with the transfer of the first leaflet onto a solid substrate by the LB method. The electrode was immersed in the PBS buffer subphase. After that, the monolayer was compressed to a surface pressure of 30 mN/m and the electrode was withdrawn above the surface of the subphase at a speed of 25 mm/min obtaining the transfer ratio of 1.0 ± 0.1. The modified electrode was allowed to dry for 30 min. Next, the second leaflet was transferred using the LS method. The lipid monolayer was compressed to a surface pressure of 30 mN/m, whereupon the electrode with the first leaflet was brought horizontally into contact with the surface of the subphase. After such procedure, bilayers with the architecture identical to the real membranes (polar heads directed outside the bilayer and hydrophobic chains towards the inside of the bilayer) were obtained and were subsequently used to perform AC voltammetry, electrochemical impedance spectroscopy (EIS) measurements and atomic force microscopy (AFM).

#### 3.2.4. AC Voltammetry (ACV)

Alternative current voltammetry measurements were conducted using CHI 750B (CH Instruments Inc., Austin, TX, USA) bipotentiostat. The Au (111) electrode with a supported lipid bilayer was used as a working electrode. During experiments, Au (111) electrode was in hanging meniscus configuration, where only surface of gold working electrode had contact with the electrolyte. The Ag|AgCl|0.1 M KCl electrode was used as a reference electrode, while the platinum wire was a counter electrode. All measurements were performed with a scan rate of 5 mV/s, AC perturbation amplitude 10 mV and the frequency set to 20 Hz. All electrochemical measurements were performed using 0.01 M PBS buffer as the supporting electrolyte. The results were presented as differential capacitance versus applied potential curves and are the average of three independent experiments. The capacitance was obtained from the in-phase and out-of-phase components of the AC current assuming a simple RC circuit.

#### 3.2.5. Electrochemical Impedance Spectroscopy (EIS)

The same potentiostat and electrode configuration as in the case of AC experiments were used during EIS measurements. Electrochemical impedance spectroscopy measurements were carried out within the frequency range of 0.1 to 1000 Hz, while the amplitude of AC perturbation was 10 mV. The spectra were collected at the potential of −0.2 V vs. Ag|AgCl, which corresponds to the potential, at which the capacitance determined from ACV measurements displayed the minimum. At such a potential, the lipid bilayer is the most densely packed and stable [71]. Obtained spectra were fitted to the equivalent circuit, where R_sol_ and R_m_ represent the resistance of solution and membrane, respectively, while Q_m_ and Q_sp_ are constant phase elements of membrane and a thin layer of water as a spacer between the bilayer and Au (111) surface. Such a circuit is commonly used to fit the EIS spectra collected for lipid membranes immobilized on electrodes [88,89,90]. The EIS results represent the average of three independent measurements.

#### 3.2.6. Atomic Force Microscopy (AFM)

AFM nanomechanical measurements were performed using Dimension Icon (Bruker, Billerica, MA, USA) in Peak Force QNM mode. In this mode, the force-distance curve is recorded on every pixel of the scanned area and z-piezo is modulated at a frequency of 2 kHz. This enables quantitative mapping of mechanical properties of the surface films. ScanAsyst Fluid probes were used for imaging with the nominal spring constant (K = 0.7 N/m). However, prior to the experiment, the probes were calibrated using the thermal tune method. The bilayers deposited on gold electrodes were analyzed based on cross-sectional profiles and adhesion mapping.

## 4. Conclusions

In this work, we have employed the Langmuir technique complimented with Brewster angle microscopy and thermodynamic analysis to compare surface properties of four different models of biological membranes containing lipid rafts. The models composed of DOPC:Chol:SM in different molar ratio were also transferred onto gold electrodes to determine their electrical properties. Based on the experimental results, it may be stated that the choice of the proper composition of the model system is crucial for providing the necessary components to ensure the coexistence of both liquid-ordered domains corresponding to lipid rafts formed by cholesterol and sphingomyelin and liquid-disordered domains due to the presence of more liquid phospholipid matrix. The main conclusions concerning the properties of the different models of raft-containing model membranes can be summarized as follows:

In the excess of DOPC, the ternary systems remain too liquid for the raft region to be stable and well separated from the matrix monolayer as shown by the isotherms and poor stability of the monolayer in the surface pressure—time measurements. This affects in turn the ability to transfer the film from the air–water interface onto the solid substrate without destroying the lipid architecture constructed at the water surface.

In the excess of Chol, the ternary layers are very solid as shown by the compressibility modulus, and the raft regions of different properties are not developed. Upon transfer to the gold surface, the solid film shows lower permeability than the one containing the excess of DOPC, which is proved by the EIS results: the phase angle at high frequencies is smaller than for the other compositions of the lipid membrane and at low frequencies the large value of phase angle indicates the lack of pores in the layer. The excess of cholesterol not only reduces the mobility of lipids in the membrane leading to the increased stiffness but also seals it, which is manifested by its lower water permeability.

In the excess of SM, the monolayers show significant changes in the structure compared to the other models, which may be ascribed to the formation of Chol:SM 1:2 molar ratio complexes. They affect the organization of the whole assembly, and the amount of the fluid matrix seems insufficient as seen by low homogeneity of the layer at the air–water interface under BAM imaging conditions. This observation is also supported by the EIS results for the films transferred on the gold substrate.

In the case of equimolar composition, the monolayer is well organized. The compression to the surface pressure of ca. 30 mN/m leads to the phase transition, during which the rafts are formed. Such model membranes remain stable in time as proved by the surface pressure-time plots and BAM images. Positive values of excess area (∆*A^Exc^*) and the excess free energy (∆*G^Exc^*) at 30 mN/m also confirm the formation of a well-organized model raft assembly with regions of different organization and membrane transport properties in the film. The separation of the microdomains was further confirmed by the behavior of the 1:1:1 layers transferred onto gold electrode, since the minima of phase angle at low frequencies on the EIS plots indicate the presence of defects on the borders of the domains as opposed to the behavior of the 2:1:1 and 1:2:1 layers, where a purely capacitive behavior of the rather homogenous films was seen.

The combination of EIS measurements and AFM imaging of the supported bilayer film composed of ternary monolayers constructed at the air–water interface under control of surface pressure measurements provide a good guide for establishing the best composition of a mixed lipid layer designed to imitate the biological membrane, e.g., for the studies of integral membrane proteins and peptides or interactions with drugs or pollutants.

## Figures and Tables

**Table 1:** Numerical values for the elements in the equivalent circuit used to model raft membranes (R_m_—resistance of membrane, Q_m_/Q_sp_—constant phase element of membrane/submembrane region, αQm/αQsp empirical constant related to the frequency dispersion; experiments were performed at the potential of −200 mV).

	R_m_ (kΩ cm^2^)	Q_sp_ (µF cm^−2^ s^α−1^)	α_Qsp_	Q_m_ (µF cm^−2^ s^α−1^)	α_m_
DOPC:Chol:SM 2:1:1	1467 ± 95	4.73 ± 0.48	0.77	6.78 ± 0.41	0.94
DOPC:Chol:SM 1:2:1	1526 ± 105	5.01 ± 0.22	0.79	6.73 ± 0.57	0.96
DOPC:Chol:SM 1:1:2	958 ± 285	5.22 ± 1.21	0.78	3.93 ± 0.69	0.94
DOPC:Chol:SM 1:1:1	1096 ± 263	3.08 ± 1.45	0.65	1.73 ± 0.42	0.97

## Data Availability

Not applicable.

## Supplementary Materials

The following are available online, Figures S1: Surface pressure—area per molecule (π-A) isotherms of (A) Sphingomyelin, Chol and DOPC monolayers formed on PBS. Insets: compression modulus vs. surface pressure plot., Figure S2: (A) Integrated charge density vs. immersion potential recorded for Au (111) electrode modified with supported DOPC:Chol:SM 1:1:2, DOPC:Chol:SM 1:2:1 bilayer, DOPC:Chol:SM 2:1:1 bilayer and DOPC:Chol:SM 1:1:1 bilayer to determine the potential of zero free charge, (B) re-scaled integrated charge density vs. immersion potential, Tables S1: Characteristic parameters of Langmuir monocompoment monolayers formed on pure PBS subphases, Table S2: Characteristic parameters of Langmuir ternary monolayers formed on PBS subphase, Table S3: Capacitance from alternative current voltammetry experiments measured at potential −200 mV and Epzfc—potential of zero free charge determined by immersion method.
