# Supplementary material for: Designing a Useful Lipid Raft Model Membrane for Electrochemical and Surface Analytical Studies
Source: Molecules. 2021 Sep 9;26(18):5483. doi: 10.3390/molecules26185483 (PMC8467995; doi:10.3390/molecules26185483)
Supplement: Supplementary file 1 [file molecules-26-05483-s001.zip › molecules-1363843-supplementary.pdf]

molecules

MDPI

Supplementary Material

Designing a useful lipid raft model membrane for electrochemical and surface analytical studies

Michalina Zaborowska[1], Damian Dziubak[2], Dorota Matyszewska[2*], Sławomir Sęk[2], Renata Bilewicz[1*]

[1] *Faculty of Chemistry, University of Warsaw, Pasteura 1, 02093 Warsaw, Poland*

[2] *Faculty of Chemistry, Biological and Chemical Research Centre, University of Warsaw, Żwirki i Wigury 101, 02089 Warsaw, Poland*

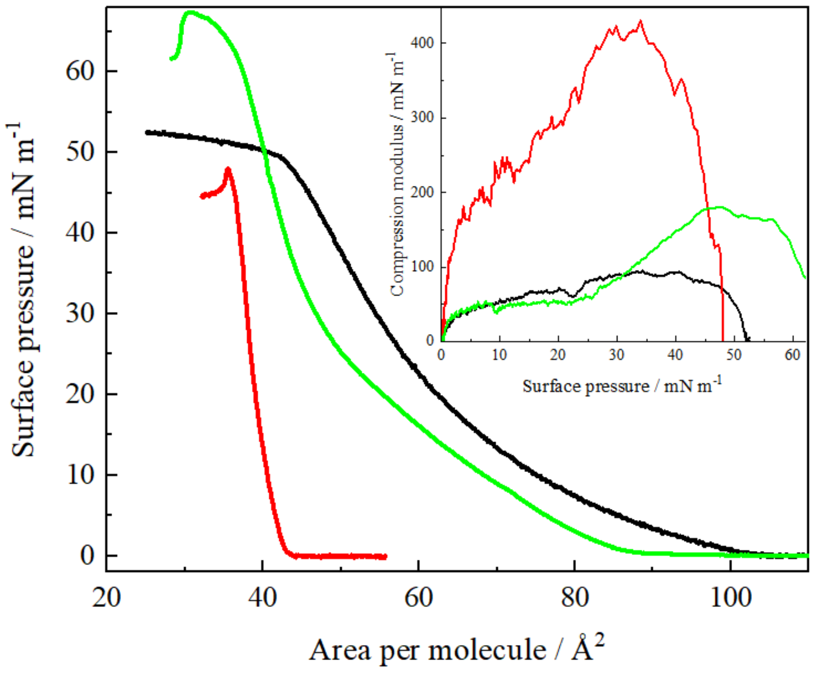

Fig.S1. Surface pressure – area per molecule (π-A) isotherms of A) Sphingomyelin (green), Chol (red) and DOPC (black) monolayers formed on PBS. Insets: compression modulus vs surface pressure plot. (T = 21 ± 1 ºC)

Table S1: Characteristic parameters of Langmuir monocompoment monolayers formed on pure PBS subphases

Subphase	A_0 (Å^2)	$A_{\pi=30\ mN/m}$ (Å^2)	A_{coll} (Å^2)	π_{coll} (mN/m)	C_s^{-1} (mN/m)
		Sphingomyelin			

PBS pH=7.4	50.7±1.0	44.3±1.0	28.5±1.2	67.5±1.7	185±6
			Cholesterol		
PBS pH=7.4	41.6±0.4	37.5±0.2	35.9±0.3	46.1±0.4	399±10
			DOPC		
PBS pH=7.4	82.8±1.0	62.1±0.5	47.2±1.1	47.8±1.1	91±5

Table S2: Characteristic parameters of Langmuir ternary monolayers formed on PBS subphase

A_0 (Å^2)	$A_{\pi=30\ mN/m}$ (Å^2)	$A_{coll/1}$ (Å^2)	$\pi_{coll/1}$ (mN/m)	$A_{coll/2}$ (Å^2)	$\pi_{coll/2}$ (mN/m)	C_s^{-1} (mN/m)
				DOPC:Chol:SM 1:1:2		
52.7±0.7	44.9±0.4	39.7±0.6	44.3±0.9	22.3±1.4	52.2±0.8	162±3
				DOPC:Chol:SM 1:2:1		
48.1±0.8	40.7±0.5	34.0±0.7	52.1±0.8	28.6±0.7	59.5±1.5	169±10
				DOPC:Chol:SM 2:1:1		
62.0±0.4	45.6±1.0	33.1±0.9	49.4±1.1	-	-	98±6
				DOPC:Chol:SM 1:1:1		
57.5±1.4	49.4±0.5	43.4±0.9	47.8±0.7	27.3±2.2	58.2±1.5	125±7

Immersion method

The potential of zero free charge (E_{pzfc}) was determined using the immersion method [1,2]. Namely, the electrode covered by the lipid bilayer was placed above the electrolyte, polarized by applying different potentials, and slowly lowered towards the electrolyte until the electrical contact was attained and the current flowing could be measured. The charge was calculated by integrating the immersion current with respect to time, and the dependence of the charge density on the applied potential was determined. The potential, where the charge was equal to zero was taken as the potential of free zero charge (E_{pzfc}). The potential of zero free charge of the unmodified - bare gold electrode was 0.146 ± 0.01 mV

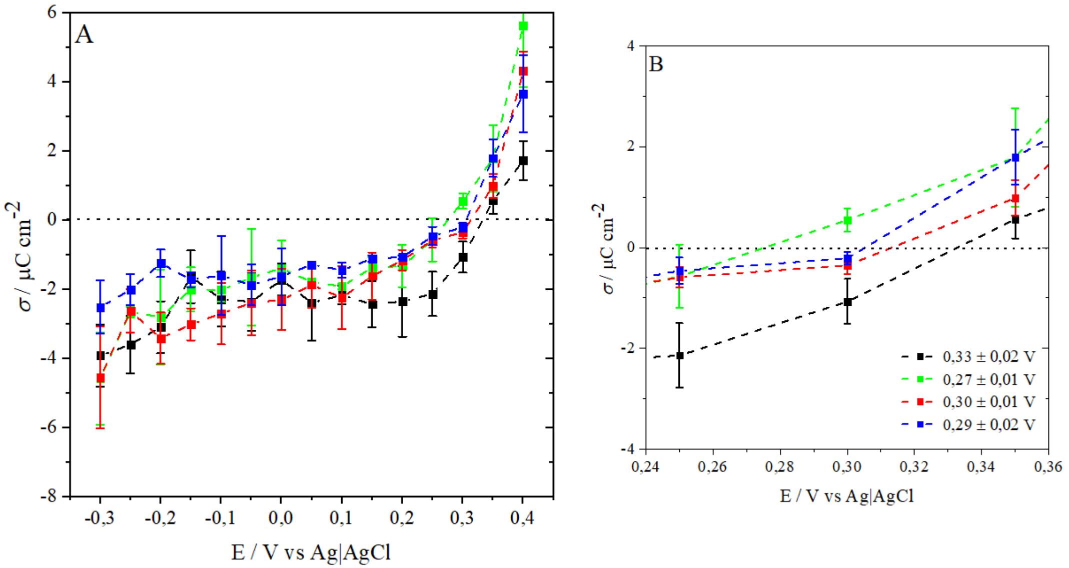

Fig. S2 A) Integrated charge density vs. immersion potential recorded for Au (111) electrode modified with supported DOPC:Chol:SM 1:1:2 (green squares), DOPC:Chol:SM 1:2:1 bilayer (red squares), DOPC:Chol:SM 2:1:1 bilayer (blue squares) and DOPC:Chol:SM 1:1:1 bilayer (black squares) to determine the potential of zero free charge, B) re-scaled integrated charge density vs. immersion potential to show better the region where the change of sign of the surface charge occurs.

Table S3: Capacitance from alternative current voltammetry experiments measured at potential -200 mV and E_{pzfc} – potential of zero free charge determined by immersion method.

$C_{ACV/E = -200\ mV}$ (µF cm^{-2})	E_{pzfc} (mV)
DOPC:Chol:SM 1:1:2	
3.93±0.69	270±10
DOPC:Chol:SM 1:2:1	
2.69±0.24	300±10
DOPC:Chol:SM 2:1:1	
3.03±0.55	290±20
DOPC:Chol:SM 1:1:1	
3.34±0.71	330±20

References

[1] U.W. Hamm, D. Kramer, R.S. Zhai, D.M. Kolb, The pzc of Au(111) and Pt(111) in a perchloric acid solution: An ex situ approach to the immersion technique, J. Electroanal. Chem. 414 (1996) 85–89. https://doi.org/10.1016/0022-0728(96)01006-6.

[2] Z. Su, J. Leitch, J. Lipkowski, Measurements of the potentials of zero free charge and zero total charge for 1-thio-β-D-glucose and DPTL modified Au(111) surface in different electrolyte solutions, Zeitschrift Fur Phys. Chemie. 226 (2012) 995–1009. https://doi.org/10.1524/zpch.2012.0280.
